# Protective Performance of Coated Reinforcement in Coral Concrete under Dry/Wet Cycling

**DOI:** 10.3390/ma16114037

**Published:** 2023-05-29

**Authors:** Hongji Cao, Qing Wu, Muhammad Akbar, Ning Yang, Zahoor Hussain

**Affiliations:** 1 College of Civil Engineering and Architecture, Jiangsu University of Science and Technology, Zhenjiang 212000, China; akbarmohammad0092@gmail.com (M.A.); yng_1972@163.com (N.Y.); 2 Institute of Mountain Hazards and Environment, Chinese Academy of Sciences, Chengdu 610041, China; 3 CCTEG (China Coal Technology Engineering Group), Coal Industry Planning Institute, Beijing 100032, China; 4 Department of Civil Engineering, Sir Syed University of Engineering & Technology Karachi Pakistan, Karachi 75300, Pakistan; dutian17@outlook.com; 5 Department of Civil Engineering, Zhengzhou University, Zhengzhou 450001, China

**Keywords:** coral concrete, chloride salt, dry wet cycle, erosion products

## Abstract

The actual protective performance of the coated reinforcement in coral concrete was investigated by measuring the chloride ion diffusion coefficient, electrochemical analysis, and numerical simulation. The test results show that the corrosion rate of coated reinforcement in coral concrete under the action of wet and dry cycles is kept at a low level, and the Rp value is always greater than 250 kΩ·cm^2^ during the test period, which is in the uncorroded state and has good protection performance. Moreover, the chloride ion diffusion coefficient D is in accordance with the power function relationship with the wet and dry cycle time, and a time-varying model of chloride ion concentration on the surface of coral concrete is established. The surface chloride ion concentration of coral concrete reinforcement was modeled as a time-varying model; the cathodic zone of coral concrete members was the most active, increasing from 0 V to 0.14 V from 0 to 20 years, with a large increase in potential difference before the 7th year, and a significant decrease in the increase after the 7th year.

## 1. Introduction

China’s marine and ocean-related businesses have experienced rapid growth as a result of the Belt and Road Initiative’s promotion of construction and the nation’s maritime defense strategy. Because of its better qualities, local availability, high efficiency, and economy, the coral reef has become an important building material for the infrastructure of offshore islands. However, the use of mixed seawater and the hostile maritime environment make steel corrosion a severe issue, reducing the service life of coral concrete structures significantly [1,2,3,4,5,6,7,8,9]. Methods for protecting marine concrete reinforcement from corrosion, such as choosing corrosion-resistant materials, adding rust inhibitors for reinforcement, applying surface coatings, cathodic protection, and electrochemical dechlorination, are equally beneficial for coral concrete [10,11,12,13,14,15,16,17]. The protection of reinforcement in coral aggregate concrete (CAC) is currently being studied by researchers. For example, Wu Changyu et al. [18,19] conducted electrochemical tests on various reinforcements in coral concrete specimens exposed for 0–270 days in the marine environment, used the linear polarization resistance method to determine the corrosion rates of various reinforcements, and discovered that 2205 duplex steel had the best corrosion resistance among various types of reinforcements. Electrochemical impedance spectroscopy (EIS) testing was used by Dabo et al. [20] to examine the impact of various reinforcement types and protective layer thicknesses on the durability performance of coral concrete. Based on their findings, they recommended designing coral concrete with a protective layer having a thickness of less than 5.5 cm. Yu et al. [21,22] found that when the minimum concrete cover thickness is 3.0 cm and the minimum concrete strength is C70, the service life requirement of 50 years can be satisfied; when the minimum concrete cover thickness is 3.0 cm and the minimum concrete strength is C80, the design service life requirement of 100 years can be met for CAC frame structures with silane coating and 304 stainless steel. According to Tang Cong’s [23] analysis of the costs of stainless steel, epoxy-coated steel, and ordinary steel over their entire life cycles, the material cost of stainless steel is roughly 4–6 times that of ordinary steel, the total cost of epoxy-coated steel’s materials and processing is roughly 2–3 times that of ordinary steel, and the costs of necessary maintenance, repairs, and anti-corrosion measures in late service come into play. The life cycle cost of stainless steel is approximately 1750 USD/ton, while the life cycle cost of epoxy-coated steel is approximately 14.00 USD/ton, which results in a 20% cost savings when using epoxy-coated steel after accounting for necessary maintenance, repair, and anti-corrosion measures.

Therefore, steel coating is more cost-effective than stainless steel reinforcement and can also be used in conjunction with other corrosion protection techniques, such as rust inhibitors, among the various methods of protecting reinforcing steel from corrosion. It also has the advantages of being simple to use and protecting the environment. However, the majority of the present research on the preservation of coral concrete reinforcement focuses on the kinds of reinforcement used and rust inhibitors, and it is not quite clear how to best maintain CAC reinforcement coatings. To increase the strength of the structure and lengthen its service life, an in-depth study of the protective qualities of reinforcing coatings in CAC is crucial.

In light of this, we conducted a test on the steel coating of coral concrete under dry and wet cycle regimes, investigated in depth the actual protection performance of steel coatings on coral concrete, and analyzed the process of coating failure by determining the chloride ion diffusion coefficient, electrochemical analysis, and numerical simulation. We sought to design a powerful analytical and computational model that can accurately depict the chloride ion infiltration process in coral concrete while protecting the reinforcing coating in a real-time marine environment.

## 2. Experimental Overview

### 2.1. Raw Materials and Mixing Ratio

**Figure 1:**
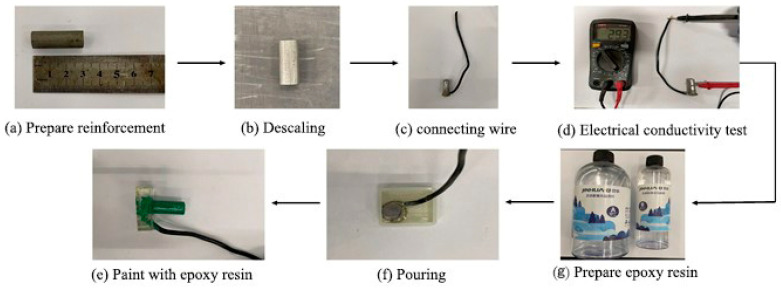
Fabrication process of reinforcement electrode.

The specimens were cubic 100 mm × 100 mm × 100 mm specimens. The coral concrete was made of Conch P-O 42.5 grade cement. The coarse aggregate was South China Sea coral reef with 5~16 mm continuous grading; the laboratory-measured apparent density of the coarse aggregate was 1865 kg/m^3^, the bulk density was 928 kg/m^3^, the compressive strength was 1.6 MPa, the porosity was 55%, the natural water content was 0.1%, and the mud content was 0.58%. Coral sand with a fineness modulus of 2.5 was used as the fine aggregate; the laboratory-measured apparent density of the fine aggregate was 2450 kg/m^3^, the bulk density was 1163 kg/m^3^, the porosity was 55%, the natural water content was 0.1%, and the mud content was 0.58%. Following ASTM D1141-2013, we artificially prepared seawater, the main components of which are shown in Table 1, incorporated with a high-performance, polycarboxylic acid-based water-reducing agent (2%). The reinforcement was a Q235 light round steel bar, 12 mm in diameter. The specific ratio of the test is shown in Table 2. Figure 1 shows the fabrication of the rebar electrodes in the experiment.

### 2.2. Experimental Protocol

The dry and wet cycles of the ocean were simulated using artificial seawater, and the dry and wet cycles were simulated once a day with a dry-to-wet time ratio of 1:1, or 12 h of immersion and 12 h of drying. After the specimens were cured, the coral concrete specimens were sealed with paraffin wax on the rest of the surface except the working surface to test at 30 days, 60 days, 90 days, 120 days, 150 days, 180 days, 270 days, and 360 days. After removing the specimens, the concrete’s surface was cured. A hand drill with a 6 mm diameter was used to drill samples at depths of 0–5, 5–10, 15–20, 20–25, and 25–30 mm. After drilling each depth, we rapidly gathered the powder and used a brush to wipe up the residual powder in the hole to prevent the mixing of powder from various depths. The samples were dried in an oven for two hours, and after cooling, 5 g was weighed and put into a beaker with 250 mL of deionized water. The magnetic stirrer was then turned on and dynamically stirred, and a rapid chloride ion content detection tester was used to measure the free chloride ion content ($C_{f}$) of the concrete powder at various depths and test times.

**Figure 2:**
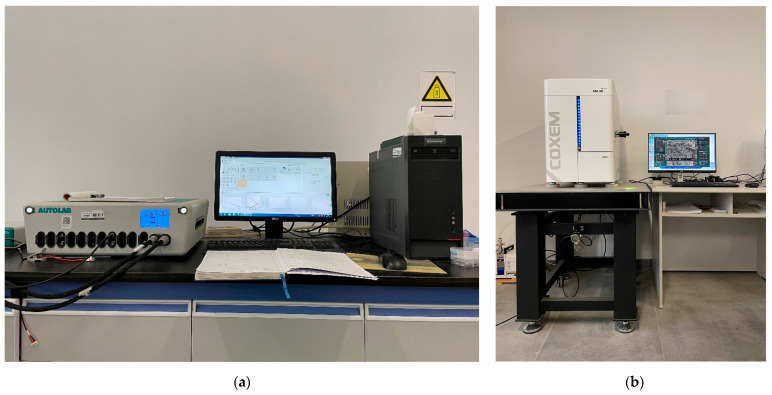
(**a**) AUTOLAB-302N electrochemical workstation. (**b**) COXEM EM-30 Plus scanning electron microscope.

After removing the coral concrete samples at 120, 180, 240, 300, and 360 days, electrochemical tests were carried out. For these tests, the saturated glycerol electrode served as the reference electrode, the coral concrete reinforcement that was to be tested served as the working electrode, and the platinum sheet electrode served as the auxiliary electrode of the three-electrode system. The components to be tested had to be immersed in saturated Ca(OH)_2_ solution for more than 8 h prior to the electrochemical test to ensure the stability of the rebar surface and the rebar/concrete interface region and to eliminate the influence of stray currents on the test results. An electrochemical workstation, model AUTOLAB-302N, made by Aptar Switzerland Ltd. (Switzerland), as shown in Figure 2, was used to analyze the specimens’ electrochemical properties at room temperature.

## 3. Experimental Results and Discussion

### 3.1. Erosion Results and Analysis under the Action of Dry and Wet Cycles

The protective layer of reinforcement had a thickness of 30 mm, and to make data handling easier, powder samples at average depths of 2.5 mm, 7.5 mm, 12.5 mm, 17.5 mm, 22.5 mm, and 27.5 mm were taken and measured to collect information on the concentration of free chloride ions $C_{f}$ at various depth segments. The correlation between the number of chloride ions and the depth of diffusion in the CAC was then examined using regression analysis in Excel. A quadratic equation was used to create a one-to-one relationship between the two variables. In order to determine the apparent chloride ion concentration $C_{s}$ in coral concrete, in general agreement with the findings of Wang Gang [1], the depth *x* = 0 was substituted into this relational equation. The coral concrete specimen’s ambient temperature remained constant during the erosion stage; hence, the only factor affecting the chloride ion diffusion coefficient (*D*) was time (*t*). Using the least squares approach and the ORIGIN software fitting, the relationship curve between the free chloride ion concentration $C_{f}$ and the diffusion depth x generated from the test may be fitted in accordance with Equation (1) to obtain the coral concrete chloride ion diffusion coefficient D.

(1) $$C_{f}=C_{0}+\left(C_{s}-C_{0}\right)\left(1-erf\frac{x}{2\sqrt{Dt}}\right)$$

where $C_{0}$ indicates the original chloride ion content in coral concrete (%); $C_{f}$ is the free chloride ion content of coral concrete (%) when time is t; $C_{s}$ indicates the free chloride ion content of coral concrete surface (%); D denotes the diffusion coefficient of chloride ions in coral concrete (m^2^/s); erf denotes the error function.

$$erfu=\frac{2}{\sqrt{\pi }}\int_{0}^{u}e^{{-t}^{2}}dt$$

The $C_{f}$ data for coral concrete were measured at 30 days, 60 days, 90 days, 120 days, 150 days, 180 days, 270 days, and 360 days under wet and dry cycle regimes, as shown in Table 3.

**Figure 3:**
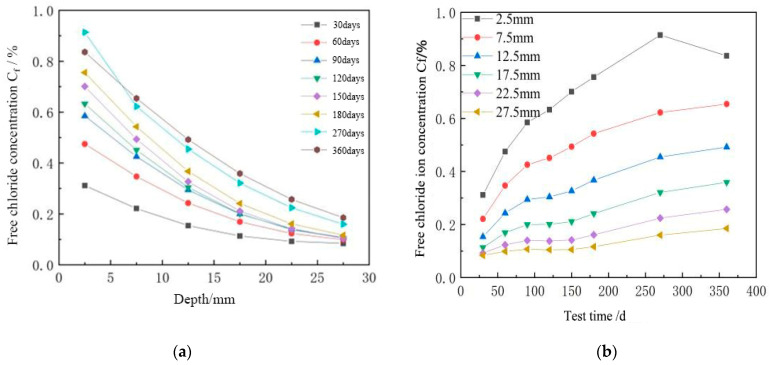
(**a**) Fick’s second rule applies to the diffusion of chloride ions in coral concrete. (**b**) Relationship between chloride ion content of coral concrete at different depths and test times.

It is evident from Figure 3 that Fick’s second rule applies to the diffusion of chloride ions in coral concrete under both the dry and wet cycling regimes. Under the dry and wet cycle regimes, coral concrete’s $C_{f}$ is inversely proportional to depth; it constantly drops with depth and stabilizes with depth. $C_{f}$ grows with the number of dry and wet cycles and reaches a maximum value of 0.1854% at 27.5 mm at 360 days. $C_{f}$ is proportional to the test time (t). Under both dry and wet cycle regimes, coral concrete’s $C_{f}$ is proportionate to depth, and $C_{f}$ rises as depth does. At various depths, the $C_{f}$ of coral concrete rose as the test duration lengthened. Among them, $C_{f}$’s growth rate was noticeably faster at depths of 2.5 mm and 7.5 mm than it was at other depths. This is primarily influenced by the wet and dry cycle regimes, where the coral concrete’s water content reaches saturation during the soaking stage, the pore solution saturation is nearly 100%, and the only effect of the chloride ions on the coral concrete’s internal diffusion process is the concentration gradient effect. The internal region of the concrete’s pore solution is still saturated at this point, even though the surface water evaporation on the concrete’s surface has accelerated the capillary process and caused the internal pore solution to start migrating outward. The chloride ions will follow the pore solution to the concrete surface. Therefore, the combined effect of surface capillary absorption and interior diffusion affects the transport process of chloride ions in coral concrete during the dry and wet cycle regimes. Water diffuses both within and outward during the drying process as a result of concentration gradients. Coral concrete develops a concentration peak as the number of wet and dry cycles increases; up to this depth, surface moisture evaporation and capillary absorption are the major processes at work in the concrete, and above this depth, diffusion is the main process at work in the concrete.

Table 4 demonstrates that the $C_{s}$ values of coral concrete increased gradually over time in both the dry and wet cycling regimes. The coral concrete’s $C_{s}$ value first increased more quickly, but as time went on, it began to develop less quickly before stabilizing. This is because as test time increases, coral concrete continues to hydrate, progressively improving the internal microstructure of the concrete, obstructing the transmission of corrosive substances, and diffusing a range of ions to the interior to remain in the concrete’s pores, increasing the resistance to permeation.

Based on the observed $C_{f}$ values, Table 5 displays the calculated diffusion coefficient D of chloride ions in coral concrete. The findings demonstrate that the chloride ion diffusion coefficient D in coral concrete tends to decrease as the test duration increases; the drop is greater in the early stages and tends to stabilize in the latter stages. Both trends are consistent with the power function relationship. The silicate cement in coral concrete gradually hydrated throughout the course of the test, producing CSH gel inside the concrete’s capillary pores. As the number of hydration products increased, coral concrete’s porosity and pore diameter dropped, which further decreased the D value of chloride ions.

The chloride ion diffusion coefficient of coral concrete under both the dry and wet cycle regimes is obtained from the results in Table 5 and is shown in Equation (2), where $R^{2}$ = 0.96.

(2) $$D={1.8274\times 10^{-10}\cdot t}^{0.639}$$

### 3.2. Electrochemical Testing

**Figure 4:**
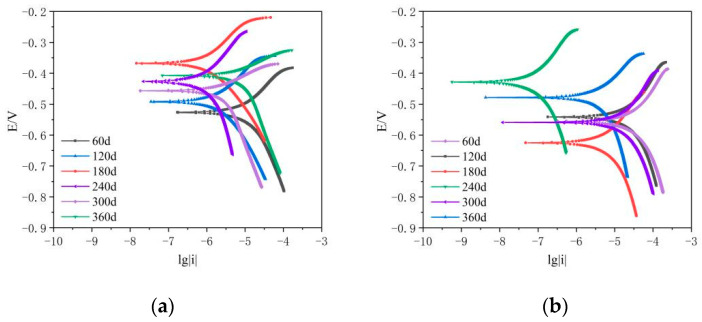
(**a**) Polarization curves of exposed steel bars with different wet and dry cycle times. (**b**) Polarization curves of coated steel bars with different dry and wet cycle times.

The polarization curves of bare and coated steel bars at various dry and wet cycle periods are shown in Figure 4a,b, respectively, and the electrochemical parameters of bare and coated steel bars at various dry and wet cycle times are shown in Table 6 and Table 7, respectively. The polarization resistance Rp values of coated steel bars were one order of magnitude higher than those of bare steel bars; however, both coated and bare steel bars’ polarization resistance Rp values declined with the lengthening of the dry and wet cycle times.

Figure 4a and Table 6 show that the exposed rebar’s Rp values were at a moderately low corrosion rate during the test period. The accumulation of corrosion products may have partially halted the progression of corrosion if the corrosion potential looked to move negatively while progressing in a positive direction. Figure 4b and Table 7 show that the epoxy-coated rebar was never corroded during the test period, with Rp values greater than 250 kΩ·cm^2^. In the dry and wet cycles after 180 days, the corrosion potential continued to move in a positive direction, indicating that the corrosion resistance of the coating improved while the corrosion rate remained low. For the epoxy-coated rebar in the dry and wet cycle at 180 days, the corrosion potential Ecorr moved in the negative direction, indicating that the corrosion resistance of coated rebar somewhat declined.

Due to the coral aggregate’s porosity and the chloride ions in the water used in the concrete mix, the surface passivation coating of exposed reinforcement in coral concrete was in a more unstable state during the 360 days wet and dry cycle test than it was in silicate cement concrete. According to the polarization resistance value, it can be seen that corrosion began to occur at 360 days while the epoxy resin coating was still intact, demonstrating a better ability to protect against corrosion. Without the protection of a coating, corrosion will activate when the surface chloride ion concentration reaches the critical value.

### 3.3. COMSOL Simulation

(a) Model Building

**Figure 5:**
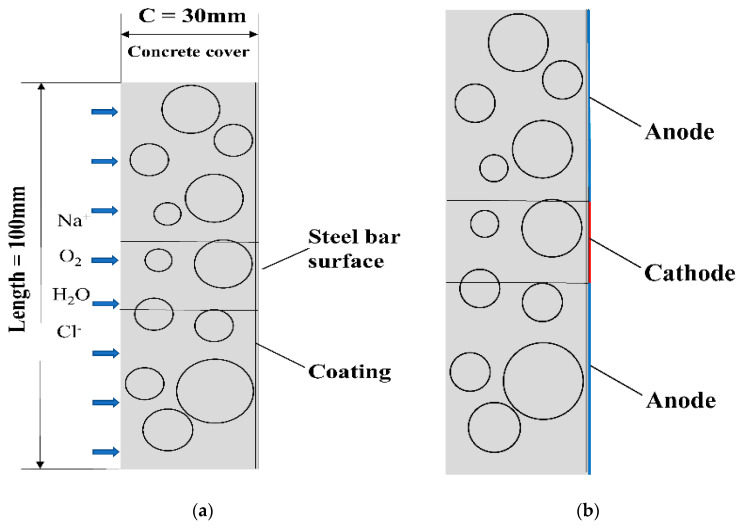
Model of coated steel in coral concrete: (**a**) simplified two-dimensional model; (**b**) cathode zone and anode zone.

This model represents the portion from the outer surface of the protective layer to the surface of the reinforcement to mimic the electrochemical corrosion on that surface. By intercepting a 100 mm long CAC part with a reinforcement coating having a thickness of 1 mm and a protective layer of 30 mm, a simplified two-dimensional model of the coated reinforcement in the CAC was developed in relation to the work of Y. D. Yan [24]. Figure 5a depicts the model, and Figure 5b depicts the separation of the cathodic and anodic zones.

(b) Model parameter setting

A cubic current distribution with a Nernst–Planck interface was used in the COMSOL model. With reference to the study by Jiuxin Fang [25], the parameters were established as displayed in Table 8. According to Wang et al.’s study [1], the specimens were simulated using a wet and dry cycle with a 1:1 wet-to-dry time ratio once each day. The simulations were used to determine the diffusion coefficients of chloride ions within the CAC. The following is a description of the chloride diffusion coefficients.

(3) $$D_{SG}={2.41676\times 10^{-10}\cdot T}^{0.67682}$$

For various times and various intrusion depths, the chloride ion content C(x,t) can be calculated using Equation (3). According to Yingying Zhang’s study [25], the diffusion coefficients of water and corrosion media within an epoxy resin covering are D = 95.6 μm^2^/d for corrosion media and T = 14 day for water to reach the substrate.

Since reactant and product concentrations are not always the same and chemical reactions often take place in non-standard states, the electrode potential is a number that changes over time. The Nernst equation is typically employed in this situation to quantitatively express the electrode potential’s magnitude. Some researchers, such as Hussain R. R. [26], employed the formula $F_{Cl}$ to describe the connection between the steel anode’s surface potential and the presence of chloride ions. The Nernst equation can be written as follows:

(4) $$\varphi_{Fe}=\varphi_{Fe}^{\theta }+\frac{RT}{Z_{Fe}F}\cdot lnC_{{Fe}^{2+}}\cdot F_{Cl}$$

(c) Boundary conditions and initial conditions

Figure 5b divides the cathode and anode areas. The only charged particles discussed in this study are those that follow the Nernst–Planck equation: Cl^−^, OH^−^, Fe^2+^, Na^+^, and Ca^2+^. For the movement of materials, the Nernst–Planck equation is employed, and for the corrosion of steel bars, the modified Tafel equation is used. An equation for the slope of the anodic Tafel reaction for steel corrosion was proposed by fitting the experimental data to rectify the anodic Tafel influence on the electrode reaction.

(5) $$\beta_{a}=\frac{4.113RT}{\alpha_{a}ZF}\cdot {C_{Cl,t}}^{-1.018}$$

where $C_{Cl,t}$ is the chloride ion content of the steel surface (%, mass as a percentage of the concrete).

(d) Boundary conditions and initial conditions

**Figure 6:**
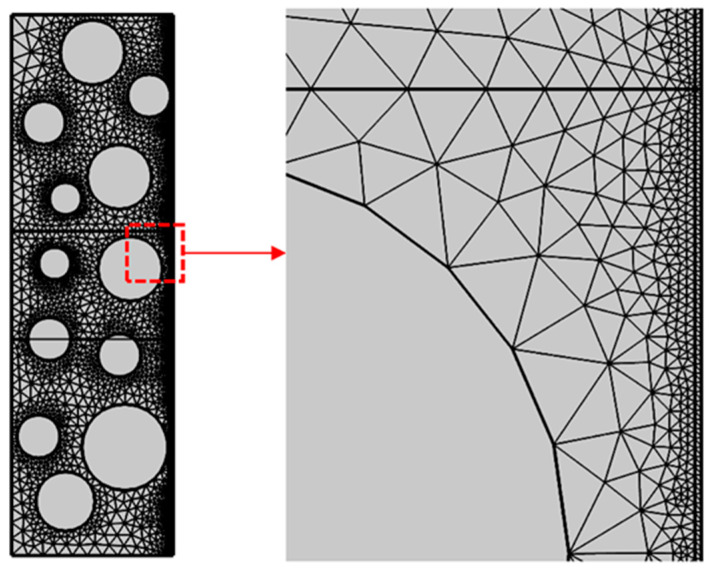
Mesh profile.

The model employs the physical field control model, sets the cell size to the standard value, and dissects the concrete, coating, and other components one at a time using the free triangular mesh. The results of the dissection are displayed in Figure 6.

**Figure 7:**
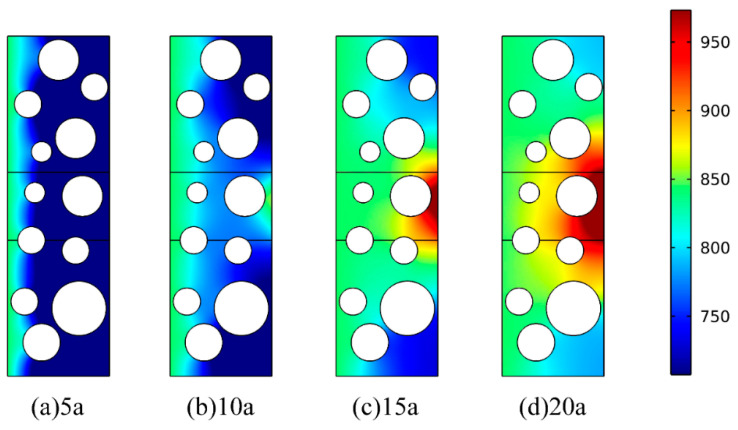
Cloud map of chloride ion concentration (“a” stands for years in this paper): (**a**–**d**).

To obtain the cloud map of chloride ion concentration distribution within the coral concrete and the potential distribution on the surface of the reinforcement, the corrosion age was set to 20 years. Figure 7 shows the cloud map of the two-dimensional chloride ion concentration distribution of CAC subjected to seawater erosion from 5a to 20a in the dry and wet cycle modes, based on the transport model simulation of Wang [1] and Yingying Zhang [25]. From the figure, it can be seen that the chloride ion concentration in the cathode area increases at 10a, and the difference between the chloride ion concentration in the cathode area and the anode area is more obvious at 20a. This is because 10a is when the corrosion medium, through adsorption, diffusion, and soluble penetration, reached the coating/metal interface, leading to the formation of the cathode area and anode area on the surface of the steel, as well as the loss of electrons from the cathode area of the steel surface corrosion microcell to form the anode, and an electrochemical corrosion reaction began to occur. However, the epoxy coating near the cathode area is firmly bonded to the surface of the rebar substrate. With no cathode peeling, delamination, or other phenomena, the corrosion media in the anode area within the epoxy coating have a small diffusion coefficient and slow transmission, and the chloride ion concentration difference gradually becomes larger.

**Figure 8:**
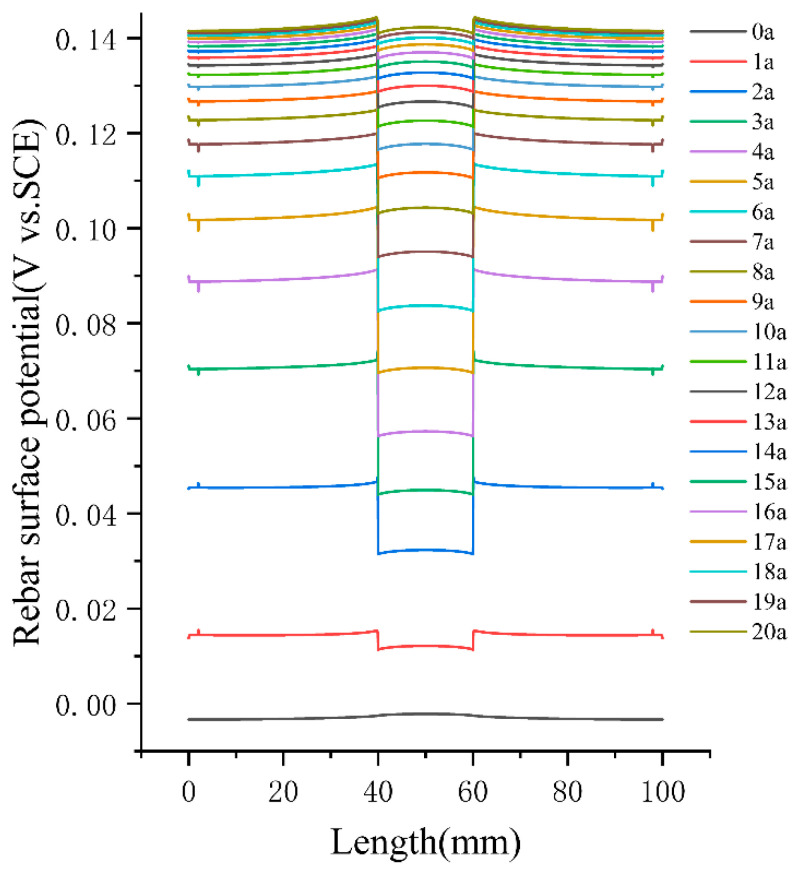
Surface potential distribution of reinforcing steel.

Figure 8 depicts the distribution of steel surface potentials. The surface forms a cathode and anode due to the uneven distribution of chloride ion concentration, which results in different potentials in different parts of the rebar. The anode area of the rebar produces active electrons, and electrons pass through the rebar to the cathode area. The cathode region rebar surface receives electrons, the rebar suffers corrosion in the electrochemical reaction of water and oxygen, and the coating is not damaged to become the anode. The potential difference between the cathode area and the anode area showed a trend of first increasing and then decreasing with time, and the conclusions obtained are consistent with the study of Juxin Fang [27].

## 4. Conclusions

To explore the corrosion of coral concrete’s coated reinforcement in the marine environment, we analyzed the variation in parameters such as chloride ion diffusion coefficient with time by measuring the chloride ion content in coral concrete at different times and depths under dry and wet cycle regimes, and we performed a qualitative analysis of the corrosion of the reinforcement by electrochemical tests. Meanwhile, the protection model of CAC-coated reinforced concrete members in natural corrosion conditions at a fine scale was established based on COMSOL, and the following conclusions were obtained.

(1) The diffusion law of chloride ions in coral concrete under the dry and wet cycle regimes is in accordance with Fick’s second law of diffusion, and a time-varying model of chloride ion diffusion coefficient in coral concrete was established based on several sets of experimental data, through which the chloride ion concentration on the surface of the reinforcement at any time under the dry and wet cycle regimes can be calculated. The Cs value of coral concrete in the dry and wet cycle regimes increased from 0.361% to 0.931% with time. The Cs value of coral concrete increased at a faster rate from 30 to 150 days, and after 150 days, the increase in Cs value continued to decrease and finally remained stable, which can help to predict the service life of coral concrete.
(2) The electrochemical test results show that the corrosion of exposed reinforcement in coral concrete started at 360 days, and that the corrosion resistance of coral concrete without anti-corrosion measures is poor. The Rp value of 3.36 × 10^6^ for coated reinforcement at the 360-day wet and dry cycle time is an order of magnitude greater than the Rp value of 3.14 × 10^5^ for bare reinforcement. The epoxy resin coating remained in the uncorroded state and showed better protection under the dry and wet cycle regimes for one year.
(3) The protection model of CAC-coated reinforced concrete members in natural corrosion conditions at a fine scale was established based on COMSOL. The cathodic zone of the member reacted the most actively, and the potential difference between the cathodic and anodic zones first showed an increasing trend and then decreased with time. It increased from 0 to 20 years from 0 V to 0.14 V, with a large increase in potential difference up to year 7 and a large decrease after year 7.

## Figures and Tables

**Table 1:** Chemical composition of artificial seawater (kg/m^3^).

NaCl	MgCl_2_·H_2_O	Na_2_SO_4_	CaCl_2_	KCl
24.5	11.1	4.1	1.2	0.7

**Table 2:** Test mixing ratio.

Concrete	Coral Aggregate	Coral Sands	Seawater	Water Reducer
557	749	749	195	11

**Table 3:** $C_{f}$ values in coral concrete under dry and wet cycle regimes.

Depth *x* (mm)	*C_f_*							
	30 Days	60 Days	90 Days	120 Days	150 Days	180 Days	270 Days	360 Days
2.5	0.3112	0.4749	0.5848	0.6326	0.7008	0.7556	0.9139	0.8363
7.5	0.2212	0.3470	0.4254	0.4511	0.4935	0.5427	0.6221	0.6541
12.5	0.1541	0.2429	0.2943	0.3043	0.3269	0.3676	0.4547	0.4921
17.5	0.1131	0.1691	0.1999	0.2010	0.2112	0.2411	0.3213	0.3587
22.5	0.0925	0.1234	0.1401	0.1380	0.1416	0.1610	0.2244	0.2571
27.5	0.0840	0.0988	0.1069	0.1046	0.1055	0.1163	0.1600	0.1854

**Table 4:** $C_{s}$ values in coral concrete under dry and wet cycle regimes.

Times/Days	30	60	90	120	150	180	270	360
Cs/%	0.361	0.544	0.670	0.730	0.812	0.869	0.914	0.931

**Table 5:** Calculation of the diffusion coefficient D values of coral concrete under dry and wet cycle regimes.

Times/Days	30	60	90	120	150	180	270	360
D (10^−12^ m^2^s^−1^)	24.145	015.156	8.946	6.743	6.543	6.106	5.844	5.133

**Table 6:** Electrochemical parameters of exposed reinforcement at different dry and wet cycle times.

Dry and Wet Cycle Time/Days	Ba/mV	Bc/mV	Icorr/μA·cm^−2^	Ecorr/mV	Rp/Ω·cm^2^
120	137.65	160.24	5.73 × 10^−5^	−491.06	5.61 × 10^5^
180	124.95	114.24	6.10 × 10^−5^	−367.39	4.25 × 10^5^
240	101.87	112.51	5.37 × 10^−5^	−428.70	3.15 × 10^5^
300	75.39	79.12	5.25 × 10^−5^	−559.00	3.19 × 10^5^
360	75.04	94.80	5.79 × 10^−5^	−477.75	3.14 × 10^5^

**Table 7:** Electrochemical parameters of coated steel bars at different dry and wet cycle times.

Dry and Wet Cycle Time/Days	Ba/mV	Bc/mV	Icorr/μA·cm^−2^	Ecorr/mV	Rp/Ω·cm^2^
120	130.98	153.17	4.84 × 10^6^	−540.87	6.33 × 10^6^
180	122.04	146.45	5.43 × 10^6^	−623.29	5.33 × 10^6^
240	114.70	129.17	6.29 × 10^6^	−429.02	4.20 × 10^6^
300	70.39	162.47	5.74 × 10^6^	−457.67	3.71 × 10^6^
120	62.53	123.14	5.36 × 10^6^	−407.74	3.36 × 10^6^

**Table 8:** Model parameter settings.

Parameters	Symbols	Value
Geometric model length (mm)	*L*	100
Protective layer thickness (mm)	*C*	30
Diffusion coefficient of Cl^−^ within CAC ($\times 10^{-12}\;m^{2}/s$)	$D_{Cl}$	Controlled by Equation (3)
Ultimate current density ($\times 10^{-2}\;A/m^{2}$)	$I_{lim}$	1.5
Anodic Tafel slope (V/decade)	$\beta_{a}$	Controlled by Equation (4)
Cathodic Tafel slope (V/decade)	$\beta_{c}$	−0.18
Anode exchange current density ($\times 10^{-6}\;A/m^{2}$)	$I_{a0}$	300
Cathode exchange current density ($\times 10^{-6}\;A/m^{2}$)	$I_{c0}$	10
Anode equilibrium potential (*V* vs. *SCE*)	$E_{eq,a}$	−0.78
Cathode equilibrium potential (*V* vs. *SCE*)	$E_{eq,c}$	0.16
OH^−^ initial concentration ($mol/m^{3}$) anode equilibrium potential	$C_{OH}$	138.4
Cl^−^ initial concentration ($mol/m^{3}$)	$C_{Cl}$	0
Fe^2+^ initial concentration ($mol/m^{3}$)	$C_{Fe}$	0
Na^+^ initial concentration ($mol/m^{3}$)	$C_{Na}$	38.9
Ca^2+^ initial concentration ($mol/m^{3}$)	$C_{Ca}$	Controlled by electrically neutral conditions
Electrolyte potential in concrete (*V* vs. *SCE*)	$\varphi_{1,0}$	0

## Data Availability

Data will be provided from the corresponding author on request.
